# Supplementary material for: The interplay between temperature and growth phase shapes the transcriptional landscape of Pseudomonas aeruginosa
Source: J Bacteriol. 2026 Mar 11;208(4):e00385-25. doi: 10.1128/jb.00385-25 (PMC13086522; doi:10.1128/jb.00385-25)
Supplement: Table S1 — Genes whose LasR regulation depends on temperature. [file jb.00385-25-s0007.pdf]

Table S1. Genes whose LasR regulation depends on temperature.

PA Locus	Gene Name	Gene Description	LasR Regulation at 37°C	LasR Regulation at 25°C	How Temperature Affects LasR Regulation
PA0024	hemF	coproporphyrinogen III oxidase, aerobic	-0.019819667	1.289756578	Negatively LasR regulated at 25°C
PA0025	aroE	shikimate dehydrogenase	0.052683089	1.12647734	Negatively LasR regulated at 25°C
PA0029		probable sulfate transporter	-1.137766372	0.744816807	Positively LasR regulated at 37°C
PA0038		hypothetical protein	-0.676269034	-1.535485651	Positively LasR regulated at 25°C
PA0043		hypothetical protein	-1.038102778	-0.166703581	Positively LasR regulated at 37°C
PA0047		hypothetical protein	0.388587597	1.475127645	Negatively LasR regulated at 25°C
PA0048		probable transcriptional regulator	-1.300288029	-0.146092638	Positively LasR regulated at 37°C
PA0049		hypothetical protein	-2.365962739	-0.503480184	Positively LasR regulated at 37°C
PA0060		conserved hypothetical protein	-0.306391201	-1.103046006	Positively LasR regulated at 25°C
PA0061		hypothetical protein	-1.156020018	-0.434606109	Positively LasR regulated at 37°C
PA0062		hypothetical protein	-1.037193126	-0.977094655	Positively LasR regulated at 37°C
PA0072	tagS1	TagS1	-1.148772338	-0.16202482	Positively LasR regulated at 37°C
PA0086	tagJ1	TagJ1	-0.14723237	1.109192444	Negatively LasR regulated at 25°C
PA0097		hypothetical protein	-0.357175623	1.216855906	Negatively LasR regulated at 25°C
PA0104		hypothetical protein	-1.504441707	-0.323706045	Positively LasR regulated at 37°C

PA0109		hypothetical protein	-1.456427165	-0.443991218	Positively LasR regulated at 37°C
PA0128		conserved hypothetical protein	1.064971095	-0.004728532	Negatively LasR regulated at 37°C
PA0129	bauD	Amino acid permease	1.05608781	-0.355484843	Negatively LasR regulated at 37°C
PA0152	pcaQ	transcriptional regulator PcaQ	-1.166602384	-0.944500494	Positively LasR regulated at 37°C
PA0159		probable transcriptional regulator	-0.520735942	-1.335171723	Positively LasR regulated at 25°C
PA0160		hypothetical protein	-0.17385085	-1.238596058	Positively LasR regulated at 25°C
PA0162	opdC	histidine porin OpdC	-0.514417287	-1.285037503	Positively LasR regulated at 25°C
PA0169	siaD	SiaD	1.626711549	-0.189970392	Negatively LasR regulated at 37°C
PA0170	siaC	SiaC	1.301206233	-0.436603986	Negatively LasR regulated at 37°C
PA0171	siaB	SiaB	2.066780482	-0.190019324	Negatively LasR regulated at 37°C
PA0172	siaA	SiaA	1.450727072	0.219287244	Negatively LasR regulated at 37°C
PA0177		probable purine-binding chemotaxis protein	-1.013597719	-0.350385342	Positively LasR regulated at 37°C
PA0189		probable porin	-0.99886364	-2.119533557	Positively LasR regulated at 25°C
PA0194		hypothetical protein	-1.528202006	-0.431579288	Positively LasR regulated at 37°C
PA0200		hypothetical protein	0.118771696	1.164022796	Negatively LasR regulated at 25°C
PA0208	mdcA	malonate decarboxylase alpha subunit	0.467838772	1.972280884	Negatively LasR regulated at 25°C
PA0209		conserved hypothetical protein	-0.009600823	1.946643017	Negatively LasR regulated at 25°C

PA0210	mdcC	malonate decarboxylase delta subunit	0.250520919	1.395039784	Negatively LasR regulated at 25°C
PA0212	mdcE	malonate decarboxylase gamma subunit	0.763131141	1.758386077	Negatively LasR regulated at 25°C
PA0213		hypothetical protein	0.595059573	4.209656225	Negatively LasR regulated at 25°C
PA0214		probable acyl transferase	1.343049379	0.719958435	Negatively LasR regulated at 37°C
PA0215		malonate transporter MadL	1.624493971	0.595815457	Negatively LasR regulated at 37°C
PA0230	pcaB	3-carboxy-cis,cis-muconate cycloisomerase	-1.129609742	-0.682410609	Positively LasR regulated at 37°C
PA0231	pcaD	beta-ketoadipate enol-lactone hydrolase	-1.335007309	-0.686009586	Positively LasR regulated at 37°C
PA0263	hcpC	secreted protein Hcp	1.780251219	-3.235073575	Mixed LasR regulation between temperatures
PA0263.1		tRNA-Arg	1.175923194	0.832378118	Negatively LasR regulated at 37°C
PA0281	cysW	sulfate transport protein CysW	1.195487103	0.269859122	Negatively LasR regulated at 37°C
PA0282	cysT	sulfate transport protein CysT	1.108979921	0.139228462	Negatively LasR regulated at 37°C
PA0283	sbp	sulfate-binding protein precursor	1.671076583	0.543674134	Negatively LasR regulated at 37°C
PA0284		hypothetical protein	1.599445082	0.19907242	Negatively LasR regulated at 37°C
PA0291	oprE	Anaerobically-induced outer membrane porin OprE precursor	1.087050415	-0.230275983	Negatively LasR regulated at 37°C
PA0298	spuB	Glutamylpolyamine synthetase	1.079068264	0.806205583	Negatively LasR regulated at 37°C

PA0309		hypothetical protein	-1.014691995	-0.92343533	Positively LasR regulated at 37°C
PA0321		acetylpolyamine amidohydrolase	0.1546683	1.295900319	Negatively LasR regulated at 25°C
PA0354		conserved hypothetical protein	-0.83097435	-1.564050799	Positively LasR regulated at 25°C
PA0369		Uncharacterized protein	-0.664658431	-1.301933785	Positively LasR regulated at 25°C
PA0397		probable cation efflux system protein	-1.056826101	-0.44483745	Positively LasR regulated at 37°C
PA0423.1	AS1974	AS1974	-1.300483465	-0.50418423	Positively LasR regulated at 37°C
PA0423.2	AS1974-shor	AS1974-shorter1	-1.298180501	-0.512820973	Positively LasR regulated at 37°C
PA0446		conserved hypothetical protein	-1.651459697	0.385544902	Positively LasR regulated at 37°C
PA0447	gcdH	glutaryl-CoA dehydrogenase	-1.221200928	0.255900437	Positively LasR regulated at 37°C
PA0451		conserved hypothetical protein	-1.50586847	0.157458234	Positively LasR regulated at 37°C
PA0467		conserved hypothetical protein	-1.04354138	-0.385621683	Positively LasR regulated at 37°C
PA0483		probable acetyltransferase	-1.343765998	-0.471005616	Positively LasR regulated at 37°C
PA0484		conserved hypothetical protein	-1.122952704	-0.270385369	Positively LasR regulated at 37°C
PA0485		conserved hypothetical protein	1.282196545	0.806748091	Negatively LasR regulated at 37°C
PA0509	nirN	NirN	0.407092482	4.550442098	Negatively LasR regulated at 25°C
PA0510	nirE	NirE	-0.460356279	4.820614432	Negatively LasR regulated at 25°C

PA0511	nirJ	heme d1 biosynthesis protein NirJ	0.128630364	4.797635162	Negatively LasR regulated at 25°C
PA0512	nirH	NirH	0.311098707	4.76874134	Negatively LasR regulated at 25°C
PA0513	nirG	NirG	-0.38856153	4.708172634	Negatively LasR regulated at 25°C
PA0514	nirL	heme d1 biosynthesis protein NirL	-0.860141254	4.75826201	Negatively LasR regulated at 25°C
PA0515		probable transcriptional regulator	-0.302641251	4.761891378	Negatively LasR regulated at 25°C
PA0516	nirF	heme d1 biosynthesis protein NirF	-0.441403412	4.62772784	Negatively LasR regulated at 25°C
PA0517	nirC	probable c-type cytochrome precursor	-0.418694362	5.165982593	Negatively LasR regulated at 25°C
PA0518	nirM	cytochrome c-551 precursor	-0.474805967	5.371409507	Negatively LasR regulated at 25°C
PA0519	nirS	nitrite reductase precursor	-0.091209123	4.718615097	Negatively LasR regulated at 25°C
PA0520	nirQ	regulatory protein NirQ	-0.041969944	2.782596219	Negatively LasR regulated at 25°C
PA0521		probable cytochrome c oxidase subunit	-0.635798204	4.341641027	Negatively LasR regulated at 25°C
PA0522		hypothetical protein	-0.576464188	4.458203356	Negatively LasR regulated at 25°C
PA0523	norC	nitric-oxide reductase subunit C	0.175033622	4.742116225	Negatively LasR regulated at 25°C
PA0524	norB	nitric-oxide reductase subunit B	-0.32238777	5.802947655	Negatively LasR regulated at 25°C

PA0525		probable dinitrification protein NorD	-1.45944272	7.540251045	Mixed LasR regulation between temperatures
PA0526		hypothetical protein	-1.114679122	2.693912679	Mixed LasR regulation between temperatures
PA0543		hypothetical protein	-1.631785023	-0.872430157	Positively LasR regulated at 37°C
PA0554		hypothetical protein	0.057453491	-1.071656568	Positively LasR regulated at 25°C
PA0574.1		tRNA-Met	1.068158721	0.690898467	Negatively LasR regulated at 37°C
PA0621		conserved hypothetical protein	1.205172428	0.594467608	Negatively LasR regulated at 37°C
PA0622		probable bacteriophage protein	1.065386979	0.667245479	Negatively LasR regulated at 37°C
PA0623		probable bacteriophage protein	1.13880713	0.619261691	Negatively LasR regulated at 37°C
PA0654	speD	S-adenosylmethionine decarboxylase proenzyme	1.761738637	0.299776517	Negatively LasR regulated at 37°C
PA0670		hypothetical protein	-1.052062344	-0.500758429	Positively LasR regulated at 37°C
PA0673		hypothetical protein	-1.135082441	0.132514645	Positively LasR regulated at 37°C
PA0676	vreR	sigma factor regulator, VreR	0.496310295	1.150669372	Negatively LasR regulated at 25°C
PA0700		hypothetical protein	-1.748572366	1.250192781	Mixed LasR regulation between temperatures
PA0704		probable amidase	-1.103732496	-0.584512628	Positively LasR regulated at 37°C
PA0709		hypothetical protein	-0.30880051	-1.097300466	Positively LasR regulated at 25°C

PA0710	gloA2	lactoylglutathione lyase	-0.23485019	-2.276673277	Positively LasR regulated at 25°C
PA0730		probable transferase	-0.019447703	-1.197296091	Positively LasR regulated at 25°C
PA0737		hypothetical protein	-0.476930799	-1.127885958	Positively LasR regulated at 25°C
PA0741		conserved hypothetical protein	1.034380651	0.419860066	Negatively LasR regulated at 37°C
PA0742		hypothetical protein	0.093980334	1.058584287	Negatively LasR regulated at 25°C
PA0743		probable 3-hydroxyisobutyrate dehydrogenase	-1.006723967	-0.145681359	Positively LasR regulated at 37°C
PA0751		conserved hypothetical protein	-1.294448927	-0.36902798	Positively LasR regulated at 37°C
PA0753		hypothetical protein	-1.825807273	-0.29423146	Positively LasR regulated at 37°C
PA0788		hypothetical protein	-1.395557895	-0.307845289	Positively LasR regulated at 37°C
PA0798	pmtA	phospholipid methyltransferase	-1.019303708	-0.529942925	Positively LasR regulated at 37°C
PA0803		hypothetical protein	-1.10311954	-0.127772881	Positively LasR regulated at 37°C
PA0806		hypothetical protein	-1.427680114	-0.115078297	Positively LasR regulated at 37°C
PA0813		hypothetical protein	-1.632599684	-0.550365402	Positively LasR regulated at 37°C
PA0836.1	P5	P5	0.344939522	1.140003543	Negatively LasR regulated at 25°C
PA0844	plcH	hemolytic phospholipase C precursor	-1.018200894	0.303008061	Positively LasR regulated at 37°C

PA0885		probable C4-dicarboxylate transporter	-1.131471776	0.905936395	Positively LasR regulated at 37°C
PA0894		hypothetical protein	-1.251169103	0.679644249	Positively LasR regulated at 37°C
PA0905.1		tRNA-Ser	1.163476309	0.255822878	Negatively LasR regulated at 37°C
PA0905.2		tRNA-Arg	1.087224221	0.274732306	Negatively LasR regulated at 37°C
PA0905.3		tRNA-Arg	1.036134305	0.280706984	Negatively LasR regulated at 37°C
PA0911	alpE	AlpE	1.343772725	0.625705634	Negatively LasR regulated at 37°C
PA0947		conserved hypothetical protein	1.116193102	0.003114435	Negatively LasR regulated at 37°C
PA0961		probable cold-shock protein	1.392340192	0.019692189	Negatively LasR regulated at 37°C
PA0964	pmpR	pqsR-mediated PQS regulator, PmpR	1.20544007	0.280681115	Negatively LasR regulated at 37°C
PA0972	tolB	TolB protein	1.052628787	0.401397622	Negatively LasR regulated at 37°C
PA0975		probable radical activating enzyme	1.453445433	0.519300642	Negatively LasR regulated at 37°C
PA0976.1		tRNA-Lys	1.379132251	-0.033066316	Negatively LasR regulated at 37°C
PA0980		hypothetical protein	-1.210746352	-0.910074088	Positively LasR regulated at 37°C
PA1013	purC	phosphoribosylaminoimidazole-succinocarboxamide synthase	1.190402366	0.269737774	Negatively LasR regulated at 37°C
PA1034		hypothetical protein	1.231262751	0.170366201	Negatively LasR regulated at 37°C
PA1051		probable transporter	0.891943961	1.204883984	Negatively LasR regulated at 25°C
PA1070	braG	branched-chain amino acid transport protein BraG	0.400069438	1.125194635	Negatively LasR regulated at 25°C

PA1071	braF	branched-chain amino acid transport protein BraF	0.710056073	1.326665408	Negatively LasR regulated at 25°C
PA1072	braE	branched-chain amino acid transport protein BraE	0.37687369	1.386432219	Negatively LasR regulated at 25°C
PA1073	braD	branched-chain amino acid transport protein BraD	0.990477288	1.175387695	Negatively LasR regulated at 25°C
PA1074	braC	branched-chain amino acid transport protein BraC	0.815469057	1.176279845	Negatively LasR regulated at 25°C
PA1075		hypothetical protein	0.891150155	1.063382992	Negatively LasR regulated at 25°C
PA1112.1			-1.016348456	-0.081104657	Positively LasR regulated at 37°C
PA1114		hypothetical protein	-0.356513502	-1.208559628	Positively LasR regulated at 25°C
PA1116		hypothetical protein	1.040897602	0.115559501	Negatively LasR regulated at 37°C
PA1123		hypothetical protein	1.483968727	0.775531735	Negatively LasR regulated at 37°C
PA1147		probable amino acid permease	-0.188634829	1.231823513	Negatively LasR regulated at 25°C
PA1166		hypothetical protein	-1.111010344	-0.545419586	Positively LasR regulated at 37°C
PA1176	napF	ferredoxin protein NapF	-1.42004259	-0.600365322	Positively LasR regulated at 37°C
PA1177	napE	periplasmic nitrate reductase protein NapE	-1.062805675	-0.476566986	Positively LasR regulated at 37°C

PA1195	ddaH	dimethylarginine dimethylaminohydrolase DdaH	0.433813382	1.221410675	Negatively LasR regulated at 25°C
PA1212		probable major facilitator superfamily (MFS) transporter	-0.930621149	-1.401838204	Positively LasR regulated at 25°C
PA1225		FAD-dependent NADPH:quinone reductase	1.527033982	-1.674965287	Mixed LasR regulation between temperatures
PA1252	dpkA	DpkA	-1.012943033	-0.712786495	Positively LasR regulated at 37°C
PA1260	lhpP	ABC transporter periplasmic-binding protein, LhpP	-0.080915131	1.864817085	Negatively LasR regulated at 25°C
PA1268	lhpA	Hydroxyproline 2-epimerase, LhpA	1.037205021	-2.949172374	Mixed LasR regulation between temperatures
PA1282		probable major facilitator superfamily (MFS) transporter	1.722170227	0.97513232	Negatively LasR regulated at 37°C
PA1287		probable glutathione peroxidase	-0.99984826	-1.273736381	Positively LasR regulated at 25°C
PA1288	odsT	oxylipin transporter	0.71318577	1.055138356	Negatively LasR regulated at 25°C
PA1300	hxuI	HxuI	-0.549119949	1.921377649	Negatively LasR regulated at 25°C
PA1312		probable transcriptional regulator	-1.182354873	-0.376045703	Positively LasR regulated at 37°C
PA1317	cyoA	cytochrome o ubiquinol oxidase subunit II	0.057830045	-1.254178574	Positively LasR regulated at 25°C
PA1318	cyoB	cytochrome o ubiquinol oxidase subunit I	0.96379718	-1.552322496	Positively LasR regulated at 25°C

PA1319	cyoC	cytochrome o ubiquinol oxidase subunit III	-0.211818401	-1.211585875	Positively LasR regulated at 25°C
PA1324.1	P9	P9	1.080458761	-0.222630155	Negatively LasR regulated at 37°C
PA1327		probable protease	-1.255838496	-0.455245902	Positively LasR regulated at 37°C
PA1349		conserved hypothetical protein	-1.584890078	-0.623115462	Positively LasR regulated at 37°C
PA1351		probable sigma-70 factor, ECF subfamily	-1.262026242	-0.009269003	Positively LasR regulated at 37°C
PA1394		hypothetical protein	-0.04169931	1.668737418	Negatively LasR regulated at 25°C
PA1407		hypothetical protein	-0.606761801	-1.47916709	Positively LasR regulated at 25°C
PA1410		probable periplasmic spermidine/putresci ne-binding protein	-0.372827009	1.235995018	Negatively LasR regulated at 25°C
PA1415		hypothetical protein	-1.209956307	0.078298151	Positively LasR regulated at 37°C
PA1418		probable sodium:solute symport protein	0.403360628	1.606916263	Negatively LasR regulated at 25°C
PA1421	gbuA	guanidinobutyrase	-0.398876464	2.24799619	Negatively LasR regulated at 25°C
PA1425		probable ATP-binding component of ABC transporter	1.217843434	0.205743914	Negatively LasR regulated at 37°C
PA1428		conserved hypothetical protein	1.03852653	0.581507985	Negatively LasR regulated at 37°C
PA1471		hypothetical protein	-1.404252963	-0.72260092	Positively LasR regulated at 37°C
PA1485		probable amino acid permease	-0.173916389	1.271400701	Negatively LasR regulated at 25°C

PA1486	bapF	beta-peptidyl aminopeptidase	0.533121669	1.255804201	Negatively LasR regulated at 25°C
PA1488		hypothetical protein	-2.785286304	0.424903011	Positively LasR regulated at 37°C
PA1504		probable transcriptional regulator	1.074620279	0.187130663	Negatively LasR regulated at 37°C
PA1511	vgrG2a	VgrG2a	0.142300963	-1.439006868	Positively LasR regulated at 25°C
PA1519		probable transporter	0.166938767	1.479336197	Negatively LasR regulated at 25°C
PA1580	gltA	citrate synthase	1.518968516	0.735887061	Negatively LasR regulated at 37°C
PA1592		hypothetical protein	-1.17292989	-0.970538494	Positively LasR regulated at 37°C
PA1593		hypothetical protein	1.017883324	-0.042819485	Negatively LasR regulated at 37°C
PA1599		probable transcriptional regulator	-1.184880935	-0.892489934	Positively LasR regulated at 37°C
PA1605		hypothetical protein	-0.857504579	-1.395250226	Positively LasR regulated at 25°C
PA1606		hypothetical protein	-0.6015786	-1.52001954	Positively LasR regulated at 25°C
PA1608		probable chemotaxis transducer	0.75075681	1.584593061	Negatively LasR regulated at 25°C
PA1620		hypothetical protein	0.069161791	3.57782156	Negatively LasR regulated at 25°C
PA1651		probable transporter	1.069304705	0.521689464	Negatively LasR regulated at 37°C
PA1673	mhr	microoxic hemerythrin, Mhr	0.818613498	1.603354007	Negatively LasR regulated at 25°C
PA1687	speE	spermidine synthase	1.226371061	-0.151303314	Negatively LasR regulated at 37°C

PA1689		conserved hypothetical protein	0.230705848	-1.421979412	Positively LasR regulated at 25°C
PA1733		conserved hypothetical protein	-1.000308083	-0.40748751	Positively LasR regulated at 37°C
PA1740		hypothetical protein	-1.154590972	1.111303789	Mixed LasR regulation between temperatures
PA1745		hypothetical protein	-1.010798363	-0.839867981	Positively LasR regulated at 37°C
PA1747		hypothetical protein	0.493936311	1.152239729	Negatively LasR regulated at 25°C
PA1757	thrH	homoserine kinase	1.40834578	0.173206679	Negatively LasR regulated at 37°C
PA1786	nasS	NasS	-1.209343512	-0.319643316	Positively LasR regulated at 37°C
PA1793	ppiB	peptidyl-prolyl cis-trans isomerase B	1.113442982	0.294860253	Negatively LasR regulated at 37°C
PA1796.2		tRNA-His	1.021230151	0.509397707	Negatively LasR regulated at 37°C
PA1796.3		tRNA-Leu	1.136648506	0.601700436	Negatively LasR regulated at 37°C
PA1796.4		tRNA-His	1.011007248	0.700362453	Negatively LasR regulated at 37°C
PA1826		probable transcriptional regulator	-1.135994337	-0.202308941	Positively LasR regulated at 37°C
PA1830		hypothetical protein	1.021036051	0.195453008	Negatively LasR regulated at 37°C
PA1831		hypothetical protein	1.101545117	0.050420811	Negatively LasR regulated at 37°C
PA1835		hypothetical protein	-1.114074637	-0.549299318	Positively LasR regulated at 37°C
PA1868	xqhA	secretion protein XqhA	-1.554466229	-0.53062911	Positively LasR regulated at 37°C
PA1880		probable oxidoreductase	-1.161258062	-0.361847815	Positively LasR regulated at 37°C
PA1898	qscR	quorum-sensing control repressor	0.132164013	-1.836671467	Positively LasR regulated at 25°C

PA1911	femR	sigma factor regulator, FemR	0.15626453	2.293392108	Negatively LasR regulated at 25°C
PA1912	femI	ECF sigma factor, FemI	0.980560656	1.263660294	Negatively LasR regulated at 25°C
PA1939		hypothetical protein	-0.486542911	-1.172809298	Positively LasR regulated at 25°C
PA1948	rbsC	membrane protein component of ABC ribose transporter	0.261417222	1.06885216	Negatively LasR regulated at 25°C
PA1973	pqqF	pyrroloquinoline quinone biosynthesis protein F	1.05407308	0.789714983	Negatively LasR regulated at 37°C
PA1980	eraR	response regulator EraR	-0.185985656	2.109406981	Negatively LasR regulated at 25°C
PA1997		probable AMP-binding enzyme	0.469373994	1.403987988	Negatively LasR regulated at 25°C
PA2024		probable ring-cleaving dioxygenase	-1.166256868	-0.293371436	Positively LasR regulated at 37°C
PA2038		hypothetical protein	1.361700076	0.164815854	Negatively LasR regulated at 37°C
PA2063		hypothetical protein	1.298060267	0.999164431	Negatively LasR regulated at 37°C
PA2074		hypothetical protein	-0.928221133	-1.893650379	Positively LasR regulated at 25°C
PA2075		hypothetical protein	-1.028123642	-0.432963584	Positively LasR regulated at 37°C
PA2085		probable ring-hydroxylating dioxygenase small subunit	-1.03150813	2.757594385	Mixed LasR regulation between temperatures
PA2109		hypothetical protein	0.858143361	1.127671832	Negatively LasR regulated at 25°C

PA2110		hypothetical protein	0.506027239	1.117905807	Negatively LasR regulated at 25°C
PA2111		hypothetical protein	0.600656407	1.068959671	Negatively LasR regulated at 25°C
PA2113	opdO	pyroglutamate porin OpdO	0.939359527	1.241176932	Negatively LasR regulated at 25°C
PA2114		probable major facilitator superfamily (MFS) transporter	0.954155658	1.138617288	Negatively LasR regulated at 25°C
PA2115		probable transcriptional regulator	1.335894652	0.322143273	Negatively LasR regulated at 37°C
PA2128	cupA1	fimbrial subunit CupA1	1.210042065	0.154697232	Negatively LasR regulated at 37°C
PA2129	cupA2	chaperone CupA2	1.500349893	-0.055591874	Negatively LasR regulated at 37°C
PA2130	cupA3	usher CupA3	1.274057171	-0.112061684	Negatively LasR regulated at 37°C
PA2131	cupA4	fimbrial subunit CupA4	1.394381698	0.491261463	Negatively LasR regulated at 37°C
PA2177		probable sensor/response regulator hybrid	-1.10209519	-0.818113981	Positively LasR regulated at 37°C
PA2181		hypothetical protein	-1.896550706	-0.934257742	Positively LasR regulated at 37°C
PA2188		probable alcohol dehydrogenase (Zn-dependent)	-0.871513477	-1.690393604	Positively LasR regulated at 25°C
PA2189		hypothetical protein	-0.978250081	-1.239318038	Positively LasR regulated at 25°C
PA2196		TetR family transcriptional regulator	-0.933383032	-1.243515849	Positively LasR regulated at 25°C
PA2197		conserved hypothetical protein	-0.775919884	-1.119501154	Positively LasR regulated at 25°C

PA2202		probable amino acid permease	2.122612913	0.563888662	Negatively LasR regulated at 37°C
PA2203		probable amino acid permease	1.996235142	0.537080719	Negatively LasR regulated at 37°C
PA2236	pslF	PslF	-1.056044293	-0.501415509	Positively LasR regulated at 37°C
PA2241	pslK	PslL	-1.225626255	-0.064778756	Positively LasR regulated at 37°C
PA2301		hypothetical protein	-3.273248089	-0.906453815	Positively LasR regulated at 37°C
PA2306	ambA	AmbA	0.145510188	-1.025923341	Positively LasR regulated at 25°C
PA2319		probable transposase	1.225647495	0.962107915	Negatively LasR regulated at 37°C
PA2338		probable binding protein component of ABC maltose/mannitol transporter	0.474229853	1.027699639	Negatively LasR regulated at 25°C
PA2339		probable binding-protein-dependent maltose/mannitol transport protein	0.72239883	2.740106835	Negatively LasR regulated at 25°C
PA2340		probable binding-protein-dependent maltose/mannitol transport protein	0.226820502	1.705918613	Negatively LasR regulated at 25°C
PA2341		probable ATP-binding component of ABC maltose/mannitol transporter	0.575242818	1.921612993	Negatively LasR regulated at 25°C
PA2348		conserved hypothetical protein	-1.746317546	3.746137905	Mixed LasR regulation between temperatures
PA2351		probable permease of ABC transporter	-1.404353642	0.637921958	Positively LasR regulated at 37°C

PA2374	tseF	TseF	-1.010534819	-0.50060406	Positively LasR regulated at 37°C
PA2375		hypothetical protein	-1.219996747	-0.753918173	Positively LasR regulated at 37°C
PA2378		probable aldehyde dehydrogenase	-1.037782587	-0.336846282	Positively LasR regulated at 37°C
PA2384		hypothetical protein	-2.192872324	-0.852252461	Positively LasR regulated at 37°C
PA2424	pvdL	PvdL	-3.275762416	-0.552329691	Positively LasR regulated at 37°C
PA2431		hypothetical protein	-1.596843467	-0.354176274	Positively LasR regulated at 37°C
PA2435		probable cation-transporting P-type ATPase	-1.149211379	-0.39405128	Positively LasR regulated at 37°C
PA2448		putative hydrolase	-2.282029602	-0.939964576	Positively LasR regulated at 37°C
PA2451		hypothetical protein	-2.900096404	0.14010675	Positively LasR regulated at 37°C
PA2465		hypothetical protein	0.576368877	-1.541272398	Positively LasR regulated at 25°C
PA2537		probable acyltransferase	0.853210567	1.822501508	Negatively LasR regulated at 25°C
PA2538		hypothetical protein	0.680917636	1.295034002	Negatively LasR regulated at 25°C
PA2539		conserved hypothetical protein	0.709238817	2.302422216	Negatively LasR regulated at 25°C
PA2541		probable CDP-alcohol phosphatidyltransfe rase	0.79951196	1.778568455	Negatively LasR regulated at 25°C
PA2559.1	srfA	SrfA	-1.155547169	-0.90968203	Positively LasR regulated at 37°C
PA2567		hypothetical protein	0.60246774	1.029002582	Negatively LasR regulated at 25°C
PA2569		hypothetical protein	-1.118625035	-0.762406555	Positively LasR regulated at 37°C

PA2572		probable two-component response regulator	-1.197229748	-0.374634805	Positively LasR regulated at 37°C
PA2575		hypothetical protein	1.258021455	0.885687262	Negatively LasR regulated at 37°C
PA2619	infA	initiation factor	1.170529913	-0.02676956	Negatively LasR regulated at 37°C
PA2629	purB	adenylosuccinate lyase	1.053892268	0.108002406	Negatively LasR regulated at 37°C
PA2636		hypothetical protein	0.563066331	2.743607421	Negatively LasR regulated at 25°C
PA2637	nuoA	NADH dehydrogenase I chain A	1.560416603	0.262809525	Negatively LasR regulated at 37°C
PA2662		conserved hypothetical protein	-0.297702077	1.602008748	Negatively LasR regulated at 25°C
PA2664	fhp	flavohemoprotein	-0.992835793	-2.058437049	Positively LasR regulated at 25°C
PA2697		hypothetical protein	-1.270706791	-0.72095389	Positively LasR regulated at 37°C
PA2701		probable major facilitator superfamily (MFS) transporter	-1.220814095	-0.470899677	Positively LasR regulated at 37°C
PA2708		hypothetical protein	-0.736814205	-1.145313455	Positively LasR regulated at 25°C
PA2763		hypothetical protein	-2.427284217	0.489523983	Positively LasR regulated at 37°C
PA2787	cpg2	carboxypeptidase G2 precursor	-1.015480889	-0.627685125	Positively LasR regulated at 37°C
PA2789		hypothetical protein	1.133252773	0.423491599	Negatively LasR regulated at 37°C
PA2799		hypothetical protein	-1.176408834	-0.305078552	Positively LasR regulated at 37°C
PA2819.1		tRNA-Gly	1.441757513	0.300209226	Negatively LasR regulated at 37°C
PA2819.2		tRNA-Gly	1.0856697	0.51755994	Negatively LasR regulated at 37°C

PA2819.3		tRNA-Glu	1.062010987	0.512724166	Negatively LasR regulated at 37°C
PA2828		probable aminotransferase	1.010014169	0.09741274	Negatively LasR regulated at 37°C
PA2829		hypothetical protein	1.738110922	0.18432867	Negatively LasR regulated at 37°C
PA2831		conserved hypothetical protein	-0.647280062	-1.222677435	Positively LasR regulated at 25°C
PA2835		probable major facilitator superfamily (MFS) transporter	-1.617518624	0.394632725	Positively LasR regulated at 37°C
PA2847		conserved hypothetical protein	-1.157151623	-0.218275097	Positively LasR regulated at 37°C
PA2851	efp	translation elongation factor P	1.242782817	0.006481752	Negatively LasR regulated at 37°C
PA2862	lipA	lactonizing lipase precursor	0.560945176	1.380524574	Negatively LasR regulated at 25°C
PA2911		probable TonB-dependent receptor	0.552741981	1.211552061	Negatively LasR regulated at 25°C
PA2920		probable chemotaxis transducer	-1.02961526	-0.721139216	Positively LasR regulated at 37°C
PA2984		hypothetical protein	-1.073236613	-0.256123017	Positively LasR regulated at 37°C
PA2991	sth	soluble pyridine nucleotide transhydrogenase	1.134102371	0.583683742	Negatively LasR regulated at 37°C
PA3009		hypothetical protein	1.172081335	0.556905203	Negatively LasR regulated at 37°C
PA3041		hypothetical protein	-1.013871513	-0.918046844	Positively LasR regulated at 37°C
PA3060	pelE	PelE	-1.007340607	0	Positively LasR regulated at 37°C

PA3063	pelB	PelB	-0.882602669	-1.026593956	Positively LasR regulated at 25°C
PA3089		hypothetical protein	-1.297629441	-0.489735647	Positively LasR regulated at 37°C
PA3133.1		tRNA-Glu	1.060494554	0.685670681	Negatively LasR regulated at 37°C
PA3141	wbpM	nucleotide sugar epimerase/dehydratase WbpM	-0.352048898	-1.078816281	Positively LasR regulated at 25°C
PA3149	wbpH	probable glycosyltransferase WbpH	-0.356187271	-1.054341216	Positively LasR regulated at 25°C
PA3162	rpsA	30S ribosomal protein S1	1.408741592	-0.102794016	Negatively LasR regulated at 37°C
PA3183	zwf	glucose-6-phosphate 1-dehydrogenase	1.013167691	0.472881971	Negatively LasR regulated at 37°C
PA3209		conserved hypothetical protein	0.751331485	1.247230342	Negatively LasR regulated at 25°C
PA3249		probable transcriptional regulator	-0.453866334	-1.288925437	Positively LasR regulated at 25°C
PA3262.1		tRNA-Asp	1.672914424	0.808354855	Negatively LasR regulated at 37°C
PA3262.2		tRNA-Val	1.636615209	0.799122468	Negatively LasR regulated at 37°C
PA3269		probable transcriptional regulator	1.026866902	0.300608519	Negatively LasR regulated at 37°C
PA3291	tli1	Tli1	0.132333916	-1.214889784	Positively LasR regulated at 25°C
PA3292		hypothetical protein	0.015644339	-1.2179584	Positively LasR regulated at 25°C
PA3311	nbdA	NbdA	-1.195949594	-0.132193764	Positively LasR regulated at 37°C
PA3315		probable permease of ABC transporter	-2.34777539	-0.209156799	Positively LasR regulated at 37°C

PA3316		probable permease of ABC transporter	-1.475642371	0.134542706	Positively LasR regulated at 37°C
PA3340		hypothetical protein	-1.231088185	0.043985048	Positively LasR regulated at 37°C
PA3360		probable secretion protein	1.287486687	0.754744454	Negatively LasR regulated at 37°C
PA3366.1	amiL	AmiL	1.636139678	0.88052104	Negatively LasR regulated at 37°C
PA3369		hypothetical protein	-0.820468033	-1.454999073	Positively LasR regulated at 25°C
PA3391	nosR	regulatory protein NosR	-0.906802925	2.728094525	Negatively LasR regulated at 25°C
PA3392	nosZ	nitrous-oxide reductase precursor	0.464047815	6.594431901	Negatively LasR regulated at 25°C
PA3393	nosD	NosD protein	-0.221230951	6.604069507	Negatively LasR regulated at 25°C
PA3394	nosF	NosF protein	0.553595986	6.870319031	Negatively LasR regulated at 25°C
PA3395	nosY	NosY protein	-1.035814114	6.960704444	Mixed LasR regulation between temperatures
PA3396	nosL	NosL protein	-4.069500523	6.207716233	Mixed LasR regulation between temperatures
PA3407	hasAp	heme acquisition protein HasAp	-1.990712864	1.119004337	Mixed LasR regulation between temperatures
PA3409	hasS	HasS	0.392565691	1.719449437	Negatively LasR regulated at 25°C
PA3410	hasI	HasI	0.467186188	1.620011269	Negatively LasR regulated at 25°C
PA3416		probable pyruvate dehydrogenase E1 component, beta chain	-1.334178049	-0.468278518	Positively LasR regulated at 37°C
PA3417		probable pyruvate dehydrogenase E1 component, alpha subunit	-1.331606328	-0.006873809	Positively LasR regulated at 37°C
PA3418	ldh	leucine dehydrogenase	-1.040394381	-0.315377746	Positively LasR regulated at 37°C

PA3426		probable enoyl CoA-hydratase/isomerase	-1.035209882	-0.530639183	Positively LasR regulated at 37°C
PA3431		conserved hypothetical protein	0.262037579	1.293801939	Negatively LasR regulated at 25°C
PA3432		hypothetical protein	0.106372187	1.327242026	Negatively LasR regulated at 25°C
PA3434		probable transposase	1.644032638	0.765616829	Negatively LasR regulated at 37°C
PA3441		probable molybdopterin-binding protein	0.807185782	1.269235659	Negatively LasR regulated at 25°C
PA3447		probable ATP-binding component of ABC transporter	-1.319741697	2.539267309	Mixed LasR regulation between temperatures
PA3450	lsfA	1-Cys peroxiredoxin LsfA	1.635089473	0.438532437	Negatively LasR regulated at 37°C
PA3457		hypothetical protein	-0.531440963	-1.088269085	Positively LasR regulated at 25°C
PA3475	pheC	cyclohexadienyl dehydratase precursor	-1.771039155	-0.719836517	Positively LasR regulated at 37°C
PA3486	vgrG4b	VgrG4b	0.047930129	-2.928305037	Positively LasR regulated at 25°C
PA3487	tle5	Tle5	-0.332792139	-2.093287525	Positively LasR regulated at 25°C
PA3488	tli5	Tli5	-0.017995788	-2.078247675	Positively LasR regulated at 25°C
PA3500		conserved hypothetical protein	0.071991141	1.017365052	Negatively LasR regulated at 25°C
PA3501		hypothetical protein	-0.162721036	1.141142133	Negatively LasR regulated at 25°C
PA3503		hypothetical protein	-0.917389402	1.508640032	Negatively LasR regulated at 25°C

PA3510		hypothetical protein	0.757446117	1.150768191	Negatively LasR regulated at 25°C
PA3516		probable lyase	-0.803913445	-1.692635684	Positively LasR regulated at 25°C
PA3535.1	reaL	ReaL	1.769220436	0.044380639	Negatively LasR regulated at 37°C
PA3568		probable acetyl-coa synthetase	0.648341008	1.341292231	Negatively LasR regulated at 25°C
PA3585	glpM	membrane protein GlpM	1.134940149	0.433256818	Negatively LasR regulated at 37°C
PA3598		conserved hypothetical protein	-0.86933794	-1.397244353	Positively LasR regulated at 25°C
PA3621.1	rsmZ	regulatory RNA RsmZ	1.114473105	0.620689552	Negatively LasR regulated at 37°C
PA3632		conserved hypothetical protein	1.065136037	0.115334679	Negatively LasR regulated at 37°C
PA3641		probable amino acid permease	1.2800681	0.468850216	Negatively LasR regulated at 37°C
PA3655	tsf	elongation factor Ts	1.02169396	-0.102637187	Negatively LasR regulated at 37°C
PA3656	rpsB	30S ribosomal protein S2	1.357704708	-0.100657962	Negatively LasR regulated at 37°C
PA3700	lysS	lysyl-tRNA synthetase	1.236577742	-0.034441634	Negatively LasR regulated at 37°C
PA3709		probable major facilitator superfamily (MFS) transporter	0.670015738	1.728170205	Negatively LasR regulated at 25°C
PA3718		probable major facilitator superfamily (MFS) transporter	0.626112786	1.077848172	Negatively LasR regulated at 25°C
PA3720		hypothetical protein	0.270112065	1.652531323	Negatively LasR regulated at 25°C

PA3723		probable FMN oxidoreductase	-1.159074493	-0.704040669	Positively LasR regulated at 37°C
PA3729		conserved hypothetical protein	1.039764983	0.739020987	Negatively LasR regulated at 37°C
PA3739		probable sodium/hydrogen antiporter	-1.056294474	-0.773074245	Positively LasR regulated at 37°C
PA3741		hypothetical protein	1.33860704	0.499567899	Negatively LasR regulated at 37°C
PA3745	rpsP	30S ribosomal protein S16	1.016325062	0.164443645	Negatively LasR regulated at 37°C
PA3757	nagR	Transcriptional regulator of N-Acetylglucosamine catabolism operon	0.755072962	1.054135052	Negatively LasR regulated at 25°C
PA3766		probable aromatic amino acid transporter	1.586445284	0.717050467	Negatively LasR regulated at 37°C
PA3820	secF	secretion protein SecF	1.124397017	-0.042767114	Negatively LasR regulated at 37°C
PA3821	secD	secretion protein SecD	1.147748954	-0.089180344	Negatively LasR regulated at 37°C
PA3822		conserved hypothetical protein	1.015162924	-0.04986583	Negatively LasR regulated at 37°C
PA3846		hypothetical protein	-1.285106295	-0.436764868	Positively LasR regulated at 37°C
PA3858		probable amino acid-binding protein	-1.356405285	-0.12818939	Positively LasR regulated at 37°C
PA3866		Pyocin S4	1.011278232	0.332250525	Negatively LasR regulated at 37°C
PA3870	moaA1	molybdopterin biosynthetic protein A1	1.640481624	0.762459283	Negatively LasR regulated at 37°C

PA3874	narH	respiratory nitrate reductase beta chain	0.571976976	1.690417805	Negatively LasR regulated at 25°C
PA3877	narK1	nitrite extrusion protein 1	0.817577888	3.545840533	Negatively LasR regulated at 25°C
PA3879	narL	two-component response regulator NarL	0.183874162	1.021421958	Negatively LasR regulated at 25°C
PA3880		conserved hypothetical protein	0.370218098	1.398625543	Negatively LasR regulated at 25°C
PA3884		hypothetical protein	0.631233753	1.007362275	Negatively LasR regulated at 25°C
PA3893		conserved hypothetical protein	0.115797197	-1.51667075	Positively LasR regulated at 25°C
PA3910	eddA	Extracelullar DNA degradation protein, EddA	0.316608989	2.045308428	Negatively LasR regulated at 25°C
PA3911		conserved hypothetical protein	-0.09331023	2.633512625	Negatively LasR regulated at 25°C
PA3912		conserved hypothetical protein	0.075192864	2.556271084	Negatively LasR regulated at 25°C
PA3913		probable protease	0.224229438	2.163455205	Negatively LasR regulated at 25°C
PA3920	copA1	CopA1	-0.830673814	-1.224711955	Positively LasR regulated at 25°C
PA3925		probable acyl-CoA thiolase	0.285499049	1.009784154	Negatively LasR regulated at 25°C
PA3953		conserved hypothetical protein	1.278970273	0.870699195	Negatively LasR regulated at 37°C
PA3957		probable short-chain dehydrogenase	-1.129987332	-0.293239792	Positively LasR regulated at 37°C

PA3962		hypothetical protein	-0.560954784	-1.137370444	Positively LasR regulated at 25°C
PA3986		hypothetical protein	-1.069251275	-0.351127784	Positively LasR regulated at 37°C
PA3993		probable transposase	1.064457034	-0.551327481	Negatively LasR regulated at 37°C
PA4005		conserved hypothetical protein	1.041964986	-0.037424636	Negatively LasR regulated at 37°C
PA4017		thioesterase	-1.179726607	-0.269899641	Positively LasR regulated at 37°C
PA4023	eat	ethanolamine transporter, Eat	0.879440072	1.760000588	Negatively LasR regulated at 25°C
PA4039		hypothetical protein	-0.807393133	-1.44931209	Positively LasR regulated at 25°C
PA4041		hypothetical protein	-1.4170441	-0.382618945	Positively LasR regulated at 37°C
PA4058		hypothetical protein	-1.251201967	0.106328094	Positively LasR regulated at 37°C
PA4067	oprG	Outer membrane protein OprG precursor	0.623879536	1.219582428	Negatively LasR regulated at 25°C
PA4072		probable amino acid permease	0.838473322	1.012772638	Negatively LasR regulated at 25°C
PA4073		probable aldehyde dehydrogenase	0.718008134	1.18209742	Negatively LasR regulated at 25°C
PA4090		hypothetical protein	1.239107401	0.48216484	Negatively LasR regulated at 37°C
PA4091	hpaA	4-hydroxyphenylacetate 3-monooxygenase large chain	0.975614038	1.07751941	Negatively LasR regulated at 25°C

PA4092	hpaC	4-hydroxyphenylacetate 3-monooxygenase small chain	0.796746486	1.139062583	Negatively LasR regulated at 25°C
PA4109	ampR	transcriptional regulator AmpR	-1.285542022	-0.256446989	Positively LasR regulated at 37°C
PA4112		probable sensor/response regulator hybrid	-1.062624189	-0.4936559	Positively LasR regulated at 37°C
PA4127	hpcG	2-oxo-hept-3-ene-1,7-dioate hydratase	1.796571608	0.654077768	Negatively LasR regulated at 37°C
PA4148		probable short-chain dehydrogenase	-1.048864834	1.320639693	Mixed LasR regulation between temperatures
PA4149		conserved hypothetical protein	0.379863303	2.303441421	Negatively LasR regulated at 25°C
PA4150		probable dehydrogenase E1 component	-0.34865464	1.669948717	Negatively LasR regulated at 25°C
PA4152		probable hydrolase	0.330911704	1.702897262	Negatively LasR regulated at 25°C
PA4153		2,3-butanediol dehydrogenase	-0.486088789	1.650588943	Negatively LasR regulated at 25°C
PA4173		conserved hypothetical protein	-0.516074946	-3.148038017	Positively LasR regulated at 25°C
PA4194		probable permease of ABC transporter	-1.583779314	0.903049939	Positively LasR regulated at 37°C

PA4206	mexH	probable Resistance-Nodulation-Cell Division (RND) efflux membrane fusion protein precursor	-1.422151852	-0.261822748	Positively LasR regulated at 37°C
PA4208	opmD	probable outer membrane protein precursor	-1.529049781	2.228974342	Mixed LasR regulation between temperatures
PA4235	ftnA	bacterial ferritin	0.465922413	1.10452429	Negatively LasR regulated at 25°C
PA4238	rpoA	DNA-directed RNA polymerase alpha chain	1.107792417	-0.053419343	Negatively LasR regulated at 37°C
PA4239	rpsD	30S ribosomal protein S4	1.183215408	0.096441818	Negatively LasR regulated at 37°C
PA4245	rpmD	50S ribosomal protein L30	1.004837408	-0.156669265	Negatively LasR regulated at 37°C
PA4246	rpsE	30S ribosomal protein S5	1.253918565	-0.121845478	Negatively LasR regulated at 37°C
PA4247	rplR	50S ribosomal protein L18	1.096897178	-0.268187487	Negatively LasR regulated at 37°C
PA4261	rplW	50S ribosomal protein L23	1.139759267	-0.158835334	Negatively LasR regulated at 37°C
PA4263	rplC	50S ribosomal protein L3	1.076893831	-0.12169116	Negatively LasR regulated at 37°C
PA4268	rpsL	30S ribosomal protein S12	1.04432772	-0.117495684	Negatively LasR regulated at 37°C
PA4270.1	P26	P26	1.30157107	-0.534495165	Negatively LasR regulated at 37°C
PA4271	rplL	50S ribosomal protein L7 / L12	1.121201665	-0.546521044	Negatively LasR regulated at 37°C
PA4272	rplJ	50S ribosomal protein L10	1.289027192	-0.454158586	Negatively LasR regulated at 37°C
PA4273	rplA	50S ribosomal protein L1	1.131723453	-0.221368024	Negatively LasR regulated at 37°C

PA4274	rplK	50S ribosomal protein L11	1.139916615	-0.151302769	Negatively LasR regulated at 37°C
PA4289		probable transporter	-1.223503748	-0.191861712	Positively LasR regulated at 37°C
PA4292		probable phosphate transporter	1.003798804	0.225397297	Negatively LasR regulated at 37°C
PA4293	pprA	two-component sensor PprA	-1.531574217	-0.747887558	Positively LasR regulated at 37°C
PA4294		hypothetical protein	-2.102823105	-0.867063021	Positively LasR regulated at 37°C
PA4296	pprB	two-component response regulator, PprB	-1.079709378	-0.568863731	Positively LasR regulated at 37°C
PA4298		hypothetical protein	-0.764656762	-2.685346428	Positively LasR regulated at 25°C
PA4299	tadD	TadD	-0.617413387	-1.299909421	Positively LasR regulated at 25°C
PA4300	tadC	TadC	-0.794264133	-1.171870148	Positively LasR regulated at 25°C
PA4301	tadB	TadB	-1.016241323	-0.859610612	Positively LasR regulated at 37°C
PA4305	rcpC	RcpC	-1.560696149	-0.686274969	Positively LasR regulated at 37°C
PA4313		hypothetical protein	-1.162105913	-0.328122086	Positively LasR regulated at 37°C
PA4343		probable major facilitator superfamily (MFS) transporter	-1.251925702	0.047811163	Positively LasR regulated at 37°C
PA4352		conserved hypothetical protein	-1.095760644	0.034273449	Positively LasR regulated at 37°C
PA4354		conserved hypothetical protein	1.003159241	0.462261997	Negatively LasR regulated at 37°C
PA4362		hypothetical protein	-1.160062716	-0.156843363	Positively LasR regulated at 37°C

PA4375	mexW	Resistance-Nodulation-Cell Division (RND) multidrug efflux transporter MexW	1.075459516	0.291077258	Negatively LasR regulated at 37°C
PA4394		conserved hypothetical protein	-0.284752947	-1.138878855	Positively LasR regulated at 25°C
PA4397	panE	ketopantoate reductase	-1.203246853	-0.203270686	Positively LasR regulated at 37°C
PA4429		probable cytochrome c1 precursor	1.010116883	0.285437582	Negatively LasR regulated at 37°C
PA4430		probable cytochrome b	1.014576056	0.262219321	Negatively LasR regulated at 37°C
PA4432	rpsI	30S ribosomal protein S9	1.461107798	0.320262014	Negatively LasR regulated at 37°C
PA4433	rplM	50S ribosomal protein L13	1.466841993	0.361614216	Negatively LasR regulated at 37°C
PA4438		conserved hypothetical protein	1.43757587	0.129135655	Negatively LasR regulated at 37°C
PA4443	cysD	ATP sulfurylase small subunit	1.262039777	0.211864901	Negatively LasR regulated at 37°C
PA4467		hypothetical protein	-1.970210497	1.365872343	Mixed LasR regulation between temperatures
PA4494	roxS	RoxS	1.046739443	0.227873158	Negatively LasR regulated at 37°C
PA4501	opdD	Glycine-glutamate dipeptide porin OpdP	0.165359097	2.163712808	Negatively LasR regulated at 25°C
PA4502	dppA4	probable binding protein component of ABC transporter	-0.18431642	1.116053402	Negatively LasR regulated at 25°C

PA4514		probable outer membrane receptor for iron transport	0.988211681	2.246157987	Negatively LasR regulated at 25°C
PA4516		hypothetical protein	-0.117101203	1.128438644	Negatively LasR regulated at 25°C
PA4517		conserved hypothetical protein	1.031501244	0.612674615	Negatively LasR regulated at 37°C
PA4519	speC	ornithine decarboxylase	-0.329783224	-1.08997688	Positively LasR regulated at 25°C
PA4535		hypothetical protein	-1.109313431	-0.475934647	Positively LasR regulated at 37°C
PA4541.3		tRNA-Asn	0.998191816	1.013148627	Negatively LasR regulated at 25°C
PA4563	rpsT	30S ribosomal protein S20	1.352617226	0.12327454	Negatively LasR regulated at 37°C
PA4567	rpmA	50S ribosomal protein L27	1.476666405	0.058033644	Negatively LasR regulated at 37°C
PA4568	rplU	50S ribosomal protein L21	1.406048641	0.003787696	Negatively LasR regulated at 37°C
PA4573		hypothetical protein	-1.308488042	-0.409089318	Positively LasR regulated at 37°C
PA4574		conserved hypothetical protein	1.672754679	-0.081738277	Negatively LasR regulated at 37°C
PA4575		hypothetical protein	-1.005844952	-0.483725657	Positively LasR regulated at 37°C
PA4583		conserved hypothetical protein	1.132408588	-0.049968933	Negatively LasR regulated at 37°C
PA4589		probable outer membrane protein precursor	-0.880133278	-1.907400094	Positively LasR regulated at 25°C
PA4608	mapZ	MapZ	-1.016753352	-0.300803479	Positively LasR regulated at 37°C

PA4610		hypothetical protein	-0.126426759	1.274155748	Negatively LasR regulated at 25°C
PA4619		probable c-type cytochrome	1.050540943	0.53596326	Negatively LasR regulated at 37°C
PA4620		hypothetical protein	0.907921053	1.003446323	Negatively LasR regulated at 25°C
PA4621		probable oxidoreductase	0.377366363	1.20133575	Negatively LasR regulated at 25°C
PA4628	lysP	lysine-specific permease	1.009467811	-0.121368192	Negatively LasR regulated at 37°C
PA4629		hypothetical protein	1.731597576	-0.063366859	Negatively LasR regulated at 37°C
PA4638		hypothetical protein	1.104227895	-0.805488304	Negatively LasR regulated at 37°C
PA4640	mqoB	malate:quinone oxidoreductase	1.179719455	0.717823702	Negatively LasR regulated at 37°C
PA4664	prmC	S-adenosylmethionine-dependent methyltransferase, PrmC	1.188631322	0.188373785	Negatively LasR regulated at 37°C
PA4665	prfA	peptide chain release factor 1	1.220916785	0.1279265	Negatively LasR regulated at 37°C
PA4669.1		tRNA-Gln	1.22683763	0.296370919	Negatively LasR regulated at 37°C
PA4671		probable ribosomal protein L25	1.591429306	-0.38713196	Negatively LasR regulated at 37°C
PA4672		peptidyl-tRNA hydrolase	1.287566134	0.008052326	Negatively LasR regulated at 37°C
PA4673		conserved hypothetical protein	1.577863348	0.208127474	Negatively LasR regulated at 37°C
PA4676	psCA3	beta-carbonic anhydrase	-1.692578882	-0.952354204	Positively LasR regulated at 37°C
PA4685		hypothetical protein	1.48654919	-0.181568714	Negatively LasR regulated at 37°C

PA4702		hypothetical protein	-1.267687546	-0.612138338	Positively LasR regulated at 37°C
PA4703		hypothetical protein	-1.879262012	-0.785893926	Positively LasR regulated at 37°C
PA4723		suppressor protein DksA	1.157688325	-0.062144124	Negatively LasR regulated at 37°C
PA4726.2	P30	P30	1.423424668	0.71521447	Negatively LasR regulated at 37°C
PA4766		conserved hypothetical protein	-1.243376848	-0.377222953	Positively LasR regulated at 37°C
PA4776	pmrA	PmrA: two-component regulator system response regulator PmrA	-1.172740538	-0.298975902	Positively LasR regulated at 37°C
PA4780		conserved hypothetical protein	-1.018540206	-0.253867362	Positively LasR regulated at 37°C
PA4785		probable acyl-CoA thiolase	-0.660749131	-1.276338987	Positively LasR regulated at 25°C
PA4786		probable short-chain dehydrogenase	-0.594246399	-1.095830582	Positively LasR regulated at 25°C
PA4787		probable transcriptional regulator	-1.118983167	0.338349764	Positively LasR regulated at 37°C
PA4788		hypothetical protein	-1.311157635	-0.914172924	Positively LasR regulated at 37°C
PA4802.1		tRNA-Sec	1.386818133	0.339404105	Negatively LasR regulated at 37°C
PA4843	gcbA	GcbA	0.792229605	1.182465638	Negatively LasR regulated at 25°C
PA4853	fis	DNA-binding protein Fis	1.40737364	0.223665848	Negatively LasR regulated at 37°C
PA4895		probable transmembrane sensor	-0.051793409	2.256584966	Negatively LasR regulated at 25°C

PA4912		branched chain amino acid ABC transporter membrane protein	1.05062993	0.511593525	Negatively LasR regulated at 37°C
PA4916	nrtR	Nudix-related transcriptional regulator NrtR	-3.138824421	-0.900665377	Positively LasR regulated at 37°C
PA4917	nadD2	nicotinate mononucleotide adenylyltransferase NadD2	-2.85785432	-0.843337334	Positively LasR regulated at 37°C
PA4918	pcnA	nicotinamidase, PcnA	1.325977439	0.899312745	Negatively LasR regulated at 37°C
PA4935	rpsF	30S ribosomal protein S6	1.024446734	-0.183436873	Negatively LasR regulated at 37°C
PA4937.1		tRNA-Leu	1.147420491	0.604690895	Negatively LasR regulated at 37°C
PA4974		probable outer membrane protein precursor	-0.628273046	-1.159190911	Positively LasR regulated at 25°C
PA5027		hypothetical protein	-1.761579659	0.092201549	Positively LasR regulated at 37°C
PA5057	phaD	poly(3-hydroxyalkanoic acid) depolymerase	-1.487372044	-0.971287437	Positively LasR regulated at 37°C
PA5076		putative amino acid ABC transporter substrate-binding protein	1.099174844	0.243241467	Negatively LasR regulated at 37°C
PA5083	dguB	Rid2 subfamily protein	0.508582444	1.110672077	Negatively LasR regulated at 25°C
PA5084	dguA	DguA	1.359841619	0.724105703	Negatively LasR regulated at 37°C
PA5087	tli5b2	type VI secretion lipase immunity protein, Tli5b2	-0.142645293	-1.074584453	Positively LasR regulated at 25°C

PA5088	tli5b3	type VI secretion lipase immunity protein, Tli5b3	0.107903703	-1.005763063	Positively LasR regulated at 25°C
PA5089	tle5b	type VI secretion phospholipase D effector Tle5b	0.081337894	-1.043097108	Positively LasR regulated at 25°C
PA5096		probable binding protein component of ABC transporter	-0.722519542	1.054034838	Negatively LasR regulated at 25°C
PA5097		probable amino acid permease	-0.224083981	1.287891667	Negatively LasR regulated at 25°C
PA5098	hutH	histidine ammonia-lyase	-0.36094981	1.193582374	Negatively LasR regulated at 25°C
PA5137		hypothetical protein	1.046525526	0.303597743	Negatively LasR regulated at 37°C
PA5138		hypothetical protein	1.100009427	0.438155765	Negatively LasR regulated at 37°C
PA5153		amino acid (lysine/arginine/ornithine/histidine/octopine) ABC transporter periplasmic binding protein	1.661624455	0.907236452	Negatively LasR regulated at 37°C
PA5154		probable permease of ABC transporter	1.750549199	0.69267987	Negatively LasR regulated at 37°C
PA5155		amino acid (lysine/arginine/ornithine/histidine/octopine) ABC transporter membrane protein	1.074195638	0.713888312	Negatively LasR regulated at 37°C

PA5162	rmlD	dTDP-4-dehydrorhamnose reductase	-1.447304091	-0.940220256	Positively LasR regulated at 37°C
PA5164	rmlC	dTDP-4-dehydrorhamnose 3,5-epimerase	-1.01705357	-0.684019304	Positively LasR regulated at 37°C
PA5181		probable oxidoreductase	-0.501255247	-1.456020329	Positively LasR regulated at 25°C
PA5194		hypothetical protein	1.026589361	0.411702838	Negatively LasR regulated at 37°C
PA5205		conserved hypothetical protein	-0.442287249	-2.151021189	Positively LasR regulated at 25°C
PA5213	gcvP1	glycine cleavage system protein P1	-1.078935199	-0.427093754	Positively LasR regulated at 37°C
PA5264		hypothetical protein	-0.101888735	-1.427192276	Positively LasR regulated at 25°C
PA5265		hypothetical protein	-0.175928181	-1.595504974	Positively LasR regulated at 25°C
PA5266	vgrG6	VgrG6	-0.391023311	-3.362498492	Positively LasR regulated at 25°C
PA5267	hcpB	secreted protein Hcp	1.075311769	-2.355044709	Mixed LasR regulation between temperatures
PA5275		conserved hypothetical protein	0.07807935	1.007419388	Negatively LasR regulated at 25°C
PA5290		conserved hypothetical protein	-0.224497112	-1.259648887	Positively LasR regulated at 25°C
PA5291	betT2	BetT2	-0.617225919	-1.066646776	Positively LasR regulated at 25°C
PA5295	proE	ProE	-0.919460343	-1.022283933	Positively LasR regulated at 25°C
PA5298		xanthine phosphoribosyltransferase	1.04021154	-0.122660668	Negatively LasR regulated at 37°C

PA5339		conserved hypothetical protein	1.073928201	0.195825828	Negatively LasR regulated at 37°C
PA5348		probable DNA-binding protein	1.072471446	0.630686909	Negatively LasR regulated at 37°C
PA5351	rubA1	Rubredoxin 1	-0.444163188	1.252122449	Negatively LasR regulated at 25°C
PA5352		conserved hypothetical protein	0.119283048	1.619936937	Negatively LasR regulated at 25°C
PA5353	glcF	glycolate oxidase subunit GlcF	-0.37735687	1.407576281	Negatively LasR regulated at 25°C
PA5354	glcE	glycolate oxidase subunit GlcE	-0.307296786	1.2981524	Negatively LasR regulated at 25°C
PA5367	pstA	membrane protein component of ABC phosphate transporter	1.132100776	0.601831485	Negatively LasR regulated at 37°C
PA5368	pstC	membrane protein component of ABC phosphate transporter	1.624483886	0.747358152	Negatively LasR regulated at 37°C
PA5394	cls	cardiolipin synthase	-0.692784634	-1.131624504	Positively LasR regulated at 25°C
PA5403		probable transcriptional regulator	1.284734039	0.275412299	Negatively LasR regulated at 37°C
PA5408		hypothetical protein	-1.529225008	0.036443216	Positively LasR regulated at 37°C
PA5409		hypothetical protein	-1.107891915	-0.151226144	Positively LasR regulated at 37°C
PA5423		hypothetical protein	-1.279028282	-0.740150275	Positively LasR regulated at 37°C

PA5426	purE	phosphoribosylaminoimidazole carboxylase, catalytic subunit	1.212568335	-0.091305567	Negatively LasR regulated at 37°C
PA5437		probable transcriptional regulator	1.269660291	0.300512901	Negatively LasR regulated at 37°C
PA5470		probable peptide chain release factor	-0.42363604	1.688147097	Negatively LasR regulated at 25°C
PA5473		conserved hypothetical protein	-1.259936854	-0.844852642	Positively LasR regulated at 37°C
PA5476	citA	citrate transporter	-0.64111253	-1.100084988	Positively LasR regulated at 25°C
PA5479	gltP	proton-glutamate symporter	1.151528323	0.357669757	Negatively LasR regulated at 37°C
PA5483	algB	two-component response regulator AlgB	-0.864741365	-1.039269153	Positively LasR regulated at 25°C
PA5484	kinB	KinB	-0.918119686	-1.162317964	Positively LasR regulated at 25°C
PA5504		D-methionine ABC transporter membrane protein	1.273285791	0.713155942	Negatively LasR regulated at 37°C
PA5505		probable TonB-dependent receptor	1.149850461	0.33097613	Negatively LasR regulated at 37°C
PA5538	amiA	N-acetylmuramoyl-L-alanine amidase	-0.130892384	-1.439129637	Positively LasR regulated at 25°C
PA5544		conserved hypothetical protein	0.383663151	1.042804601	Negatively LasR regulated at 25°C
PA5568		conserved hypothetical protein	1.328945443	0.00947819	Negatively LasR regulated at 37°C

PA5569	rnpA	ribonuclease P protein component	1.019380067	0.108734025	Negatively LasR regulated at 37°C
PA5570	rpmH	50S ribosomal protein L34	1.168660646	0.012810537	Negatively LasR regulated at 37°C
